# Rethinking approaches of science, technology, and innovation in healthcare during the COVID-19 pandemic: the challenge of translating knowledge infrastructures to public needs

**DOI:** 10.1186/s12961-021-00760-8

**Published:** 2021-07-21

**Authors:** Renan Gonçalves Leonel da Silva, Roger Chammas, Hillegonda Maria Dutilh Novaes

**Affiliations:** 1 Health Ethics and Policy Lab, Department of Health Sciences and Technology, Institute of Translational Medicine, Eidgenössische Technische Hochschule ETH Zürich, Zürich, Switzerland; 2 Centro de Investigação Translacional em Oncologia, Faculdade de Medicina, Instituto do Câncer do Estado de São Paulo, Universidade de São Paulo, São Paulo, Brazil; 3 Departamento de Medicina Preventiva, Faculdade de Medicina, Universidade de São Paulo, São Paulo, Brazil

**Keywords:** Science, Technology, Innovation, Public health, Health research, Health policy, Politics of knowledge in health, Precision medicine, COVID-19 pandemic

## Abstract

The coronavirus disease 2019 (COVID-19) outbreak made it clear that despite the potential of science, technology, and innovation (ST&I) to positively impact healthcare systems worldwide, as shown by the rapid development of SARS-CoV-2 test diagnostics and new mRNA vaccines, healthcare stakeholders have faced significant challenges in responding to the crisis through well-integrated ST&I-oriented health initiatives and policies. Therefore, the pandemic has mobilized experts, industry, and governments to evaluate alternative trajectories to promote a more efficient dialogue between ST&I and public health. This article presents a critical thinking about the contemporary asymmetries in the technical and political infrastructures available for particular approaches in ST&I in health, such as precision medicine, and for public health systems worldwide, uncovering a persistent gap in the translation of knowledge and technologies to adequately coordinated responses to the pandemic. We stimulate the understanding of this process as a matter of translation between platforms of knowledge and policy rationales shaped by different institutionalized frames of organizational practices and agendas. We draw attention to the need to strengthen governance tools for the promotion of ST&I as a strategic component of the post-pandemic agenda in public health, to prepare societies to respond efficiently to future emergencies.

## Introduction

The coronavirus 2019 (COVID-19) pandemic has changed how we understand and approach problems in science, technology, and innovation (ST&I) in health in contemporary society. The current situation has produced specific demands for health systems, and an inconvenient paradox has become visible: we have never had such a supply of qualified scientific and technological knowledge infrastructures in health and biomedical sciences, but at the same time a viable translation of this knowledge to public health systems has shown itself flawed and inefficient. This paradox raises questions about what has brought us to this inconvenient reality, and the importance of paying greater attention to the mechanisms of governance and implementation of ST&I in public health in a more systemic way.

Inevitably, it pushes us back to reflect about how health research has been institutionalized by contemporary science and technology policy (S&TP) agendas and regimes of knowledge in biomedical sciences. Over the past few decades, governments, top-ranked academic institutions, and the S&TP around the world have funded biomedical research with a focus on expanding our understanding of the processes of health, illness, and medicalization through the introduction of new gene and immune cell therapies to molecular levels [1]. Although the anticipated impacts of genomic sciences have not yet been fulfilled, their implementation has been promoted as a potential transformative agent in healthcare, leading to significant impacts in public health systems internationally [2, 3]. The utilization of molecular data as a basis for clinical diagnosis and practice has led to the proposition of fields such as so-called precision medicine (PM), a name advocated by a wide range of United States experts, entrepreneurs, and politicians since 2015, which has led to a significant push to reorganize interests and political agendas in academia, governments, and industry [4].

PM is an example of political viability in the making for ST&I-oriented agendas in health. The generous availability of resources accumulated and used for PM have stimulated stakeholders in healthcare to pursue new biotechnologies and personalized therapies making use of high-tech-based machines from well-furnished and expensive molecular biology laboratories. It has made possible the creation of a new research infrastructure, leading to fruitful spillovers into the international research on non-transmissible chronic, genetic, and autoimmune diseases [5].

The drive toward PM has built itself as an achievement of ST&I incursions into health in the recent years, capable of attracting media attention and large public and private investments. However, since knowledge is a human entrepreneurship that is not played in a political vacuum—that is, it results from choices about what research to undertake, and what research to leave undone [6]—it is particularly relevant to ask why this technical and political infrastructure has not been properly used by public health providers as a viable knowledge platform to reduce the negative impacts of the pandemic. Recent work has shown that the international PM community did not respond to the COVID-19 pandemic with practical solutions or clear political positioning in favour of national public health policies [7]. Thus, the recent impact of ST&I infrastructures on public health in general, and of PM infrastructures on the COVID-19 outbreak responses specifically, has not yet been determined, and it is clear that its potential should be explored through systematic multidisciplinary research as a tool of preparedness for future emergencies and better use of ST&I resources and capacity.

This article presents a critical thinking about the contemporary asymmetries in the technical and political infrastructures available for particular approaches in ST&I in health, such as PM, and for health policies and systems worldwide, uncovering a persistent gap in the translation of knowledge and technologies into adequately coordinated responses to the pandemic. Through the multidisciplinary theoretical background, we stimulate the understanding of this process as a matter of translation between platforms of knowledge and policy rationales shaped by different institutionalized frames of organizational practices and agendas.

The paper is organized as follows. The first section, “ST&I in healthcare during the COVID-19 pandemic”, traces introductory remarks regarding the importance of thinking critically on the global asymmetry of infrastructures of ST&I in health and the importance of the communication between that and public health agendas as a lesson learned during the pandemic. In the following section, “Translating precision medicine infrastructures to public health: a difficult challenge”, we present PM as an ST&I approach in health in which clinical and healthcare delivery operates out of the technical and political toolbox of public health. To illustrate, after pointing out some characteristics of PM, we show how it becomes explicit in the incorporation of technologies in health systems—typically misinterpreted as an ultimate way to approximate ST&I and public health. Next, the third section “Improving governance of ST&I for public health needs as a post-pandemic outcome” addresses the necessity of strengthening governance tools for the promotion of ST&I as a strategic component of the post-pandemic agenda in public health, particularly inspired by recent achievements reported in the literature on the politics of science and technology in health, and implementation sciences (IS). In the conclusion section, we call attention to the urgence of an academic multidisciplinary research agenda that looks for ways to shorten the distance between platforms of knowledge and rationales of decision-making in PM and public health, with the potential to be reached by the institutionalization of suitable political and cultural frames that facilitate dialogue and bridge common solutions between ST&I-oriented health approaches and the public health policies and systems.

## ST&I in healthcare during the COVID-19 pandemic

The rapid spread of the severe acute respiratory syndrome coronavirus 2 (SARS-CoV-2) pandemic has given rise to novel elements for the study of cultural implications of agendas of ST&I in contemporary healthcare. During the COVID-19 pandemic, societies have demonstrated great fragility in translating science and technology infrastructures into more efficient public health solutions to mitigate the disease around the world [8]. It revealed strong asymmetries in the global platforms of biomedical and health knowledge, as well as an inefficient system of governance and communication between approaches of ST&I and public health.

According to Jasanoff and colleagues (2021), during the novel coronavirus outbreak, decisions that were made in the past positioned some countries better than others in terms of their capability to respond to the crisis. It has been a particular way to approach the development of knowledge infrastructures in health in the field of science and technology studies (STS), and researchers in this field have been paying attention to the evolution of the pandemic responses from an international comparative perspective, taking into account the role of the political and knowledge platforms to respond to the rise in infections [9]. Countries such as Japan, South Korea, and Singapore have become leaders in health innovation over the past few decades, producing new technologies and devices, but have also experienced a positive movement of policy-making directed at facilitating and fostering research and technological development regionally [10]. Governments from countries such as the United States and United Kingdom mobilized their national systems of innovation to produce new diagnostics, devices, and technologies, but delayed in adequately producing and delivering molecular diagnostics. These countries have a sophisticated health innovation infrastructure and should have been capable of large-scale production of molecular tests in order to monitor the escalation of the pandemic and propose adequate public policies.

However, the availability of well-funded infrastructures of ST&I in health in certain countries cannot be considered as the only aspect associated with better COVID-19 responses; the inequality between those platforms around the world produced additional complications in the coordination of inter- and intra-national public health providers in their capacity to control the rapid spread of the virus. In the pandemic, even well-prepared societies faced substantial challenges to achieving an optimal encounter between ST&I and public health interests over the course of the outbreak. Countries have failed due to several factors unrelated to available scientific knowledge, such as the degree of investment in sustainable technological development, public healthcare, and health policies in general [11], resulting in ineffective systems for the prevention, vigilance, and monitoring of the spread of the novel coronavirus.

In 2020, despite the geographically unequal delivery, the unprecedented rapid technological development of several COVID-19 vaccines was proven to move in the opposite direction. It demonstrated the great capacity of scientific knowledge-based solutions to respond efficiently to the outbreak, with a strong impact on global public health strategies—an example of what is possible when a sociopolitical deal is guaranteed by governments, industry, and the global scientific community [12].

The pandemic showed that, under extreme conditions, scientists, companies, and the public sector might have the ability to implement actions addressing the provision of services and products for public health, as discussed by da Silva and colleagues (2020), with university participation in the production of molecular diagnostic tests such as reverse transcription polymerase chain reaction (RT-PCR) for the novel coronavirus in Brazil as one example [13]. This initiative faced important management sustainability challenges, but stimulated an approximation of the local scientific community around this issue [14].

Research policies in Europe, the United States, and developing countries have recognized that knowledge does not create a social impact by itself, and that all types of research are relevant, from “theoretical” to “applied”. Research agencies must use strategies that bring together a larger and more heterogeneous group of agents from different economic and social sectors, who are essential to the process from knowledge to utilization. In the health sector this has proven to be especially relevant, due to the active participation of health systems and services, health policies, health professionals, patients, and populations in this market [15].

The debate on the development of socially responsible regimes of ST&I in health has been the object of intense investigation. Researchers have recommended that academic research institutions, companies, and funding agencies take into consideration the new demands of democratic societies, in terms of financial and environmental sustainability and social equity, in the production and diffusion of novel knowledge and technologies. Institutions must, hence, commit to ethical guidelines and best practices in research, and effectively respond to fast-growing denialism, fake news, digital platforms, and other types of political and cultural production of ignorance and disinformation [16].

New expertise from interdisciplinary fields has been required to understand that the lack of dialogue and integration between ST&I platforms and agendas of public health might be a problem played out in cultural arenas. Researchers such as Parthasaraty (2020) [17] have addressed the societal implications of the problems related to ST&I in health during the pandemic from a political and policy perspective. In the same direction, Cruz and colleagues (2020) advocate that S&TP must act to ensure more responsible, equitable, and socially inclusive technological development in the coming years [18].

Since the aim of this paper is to provide a critical thinking about the contemporary asymmetries in the technical and political infrastructures available for particular approaches in ST&I in health and for the public health systems worldwide, we thus want to illustrate this by presenting the field of PM as one important approach in terms of its recent historical capacity to mobilize resources and technical and material knowledge infrastructures in the healthcare sector. Years of robust investments in labs, research facilities, projects, and consortia in PM improved the technical capacity of some countries in tailoring rapid development of medical devices, drugs, and sophisticated mathematical predictive models applied to the forecasting and management of the infections [19]. Then, based on the example of PM, we show how the global emergency of the coronavirus disease uncovers a persistent gap in the translation of knowledge and technologies into adequate responses to the pandemic. Despite the global stock of ST&I infrastructures in healthcare, it was inconveniently unavailable for solving public health needs, for reasons we will try to touch on introductorily in this work.

## Translating PM infrastructures to public health needs: a difficult challenge

PM has been largely described in the literature as an approach to the social organization of medicine, in particular addressing challenges of this field at the level of the physician–patient relationship. A team of experts from Columbia University’s Precision Medicine and Society Program in New York City recently addressed this issue in *Genetics in Medicine* (2018):

PM is part of a longstanding attempt to reorient medical diagnosis and treatment to take advantage of genomics research and other approaches leveraging big data, such as electronic medical record research and crowd-sourced health tracking. These efforts are progressively elaborating an increasingly coherent vision of a different kind of medicine. [20]

It has had unprecedented implications in the arena of medicine and healthcare, pushing institutions to rethink healthcare practices, medical education, and the limits of introducing those technologies in physicians’ daily work. But here we go in a different direction, calling attention to the aspect of PM as an ST&I approach in health [21]. Therefore, a possible way to describe this approach is that it guides academic activities and narratives of the scientific community, *policy-makers*, and business agendas, fostering the development of new research, health products, and services tailored to the individual. It is based on intensive knowledge production in molecular biology, bioinformatics, genomics, data science, machine learning, and artificial intelligence-based tools, producing diagnostics, inputs, drugs, and management systems to prevent, monitor, and treat users, clients, and patients [22].

PM can also be understood as a bandwagon tool in the sciences, being introduced consistently by the broad community of molecular biology, epidemiology, and translational sciences as a political flag to claim higher investments but also to improve interdisciplinarity in biomedicine around the world [4]. Data from the Institute for Scientific Information Web of Science (ISI WoS) Core Collection illustrate the growth of scientific production related to PM over recent decades, with a total of 22,524 articles by 2019. The decade of 2010 to 2019 accounted for 88.81% of all articles identified in the database, while the period from 2015 to 2019 alone had 69.23% of all publications, evidencing a growing interest in this research topic in recent years.

Experts from governmental boards and international associations such as the Centers for Disease Control and Prevention (CDC) Genomics and Precision Health and Precision Medicine Coalition advocate that this multidisciplinary and multisectoral approach has practical implications in the technical, intellectual, and political platforms of health research and practice [23]. However, when we analyse the recent trajectory of this movement internationally, we see central aspects of it built and being played far from the reach and scope of public health interests [24].

Although tailored medicine has been valued in academic research for some time, since 2010 this subject has gained wide interest and space in S&TP, media, business, and political discourse. The development of precision high-tech-based goods has become a popular pursuit in both Western and Eastern societies [25], especially when then United States President Barack Obama launched the Precision Medicine Initiative (PMI) in 2015. It had a strong symbolic impact in the international scientific community and for its global governmental and private stakeholders. In 2016, the United States Senate approved a budget of US$300 million for PMI for the 2017 fiscal year, $100 million more than in the previous year. The budget for all ongoing PM-based projects under the National Institutes of Health (NIH) umbrella, such as research on Alzheimer’s and rare genetic diseases, reached US$34 billion [26]. Investments in research and development (R&D) were directed to novel biotechnologies for human health, and new university–industry partnerships emerged to design and produce innovative clinical diagnostics, medical devices, data-based management systems for hospitals and health professionals, and off-the-shelf products. An important aspect of PM is its focus on the design of technologies and solutions to problems in rare, chronic, noncommunicable diseases, presenting a new package of problems to be solved to the public and private healthcare systems.

Although this is an emerging approach which has encountered challenges in its establishment, profits and commercial outcomes in this field are evident, and it gives an idea of the constraints faced by low- and middle-income countries' economies in accessing developments in this sector. According to Global Market Insights, the market for PM stood at around $57 billion in 2019, with predicted growth of 11% per year for the period 2020–2026. It is estimated that more than 40% of the sector is concentrated in North America around university-related biotechnology hubs. The year 2018 was a hallmark for this sector, as 25 products based on PM were approved by the Food and Drug Administration (FDA), with an estimated 40% of all new products in the pharmaceutical industry having a PM origin by 2025 [27].

But to what extent has this new technical and knowledge platform contributed to the improvement of infrastructures, capabilities, and preparedness of public health systems? A pragmatic way to address this is to know a bit more about current challenges for these technologies in reaching users of public health systems. According to Patricia Danzon, although innovation in health favours the supply of improved drugs and other products from the healthcare market, the introduction of new knowledge-intensive technologies (such as diagnostics and biological therapies based on genomics and molecular biology) has been one of the main factors contributing to increased healthcare expenditures for countries and families, impacting the financial sustainability of health systems worldwide [28, 29].

Not only has the fiscal unviability of PM technologies and healthcare solutions been gaining attention in recent years as a topic in official boards of experts of international organizations, but the role of innovations themselves as societal phenomena in replacing political arenas and organizational cultures has also come to the fore. In 2016, after a public debate on “Disruptive innovation: Considerations for health and health care in Europe”, the Expert Panel on Effective Ways of Investing in Health (EXPH) signed a final opinion published by the European Commission (EC).

The Expert Panel understands “disruptive innovation” in health care as a type of innovation that creates new networks and new organisational cultures involving new players, and that has the potential to improve health outcomes and the value of health care. This innovation displaces older systems and ways of doing things. The Expert Panel conceptualizes disruptive innovations as complex and multidimensional, categorizing five dimensions of disruptive innovations: typology of business model, fluency of implementation, health purposes, fields of application and pivoting values. The Expert Panel identified five strategic areas for disruptive innovation: translational research, access to new innovative technologies, precision medicine, health and care professional education and health promotion. [30]

The Expert Panel then recalled that disruptive innovations, as PM most certainly is, imply a certain degree of competition and tension with existing technologies and organizational cultures, reshaping older systems and ways of doing things, and that the overall sustainability of the healthcare system must always be considered. However, experts usually overestimate the promise and scope of technological innovations in healthcare, making recommendations to public health providers and hands-on stakeholders that are too general, and only rarely move toward a practical framework of effective actions [31, 32].

The poor adhesion to those recommendations has made the divergence of rationales between PM and public health explicit especially in the context of poor resource availability for both ST&I in health and the functioning of the health systems. In developing countries such as Brazil, for instance, this debate was recently raised by Novaes and Soárez (2019) in discussing the challenges of incorporating so-called orphan drugs for treatment of rare diseases in the Brazilian National Health System (Sistema Único de Saúde, SUS) [33].

Low- and middle-income countries also possess significant budget restrictions and large regional asymmetries in the delivery of health technologies and inputs that hinder the incorporation and measurement of the results of these initiatives—which may have a permanent fiscal impact on health systems [34]. Several public health analysts go in a similar direction: Jorge Iriart (2019) advocates that the introduction of PM-based technologies in healthcare systems could increase inequality in access to health [35]; Rey-López et al. (2018) stress the importance of critically assessing the role of PM in healthcare development, and they consider that the emphasis on technology-based solutions to prevent and treat disease individually disregards the fact that public health depends essentially upon favourable social conditions [36].

It is also an issue which has been studied by researchers and analysts in developed countries like the United States, despite the active role of the stakeholders in this country in the fields of ST&I in health and its central position in leading initiatives and disruptive innovations in PM. Lindsey Konkel (2020) recently called attention to the challenges of current research agendas in PM, and the limited potential of those technologies to be delivered broadly to the public. Interviewed by the author, Professor Esteban Burchard, MD, PhD, the Hind Distinguished Professor of Pharmaceutical Sciences and co-director of the University of California, San Francisco (UCSF) Center for Genes, Environment, and Health, gave his opinion that “in 30 years, health care will look really good for some people and really bad for others, simply because modern scientific advances have not been applied to all populations equally” [37], in a clear reminder that PM should not be taken for granted as the future of healthcare even in high-income societies.

The previous examples show how difficult it is to approach the topic of merging knowledge platforms and rationales in PM and healthcare. Traditionally, the challenge of putting this agenda into practice places technological innovation and the promotion of public health in supposedly opposing fields—something that, at the same time, reinforces the idea that PM is only useful in the scope of discourse, and the belief that ST&I infrastructures would be available only for business ventures in the health sector.

The pandemic made us critically rethink this relationship, as knowledge and practices in ST&I in health in general, and those adopted in PM specifically, could be applied to reduce the negative impacts of the pandemic, guiding more precisely the actions in public health based on previously accumulated knowledge.

## Improving governance of ST&I for public health needs as a post-pandemic outcome

Why, then, has the increased investment and research in PM over the past decades not led to improved public health with the same dynamism? The answer lies in the fact that the limited translation of ST&I infrastructures and knowledge platforms from PM into public health agendas stems from, among other factors, the lack of governance tools, institution-building, and political coordination between different stakeholders of ST&I and of the public health systems and services. A possible way to address this challenge is found in the literature on the politics of science and technology in health, and its relationship with the logics of the healthcare systems per se [38]. This interdisciplinary domain is moving toward understanding the translation of knowledge platforms as a set of sociotechnical processes played in specific political arenas, in which the coproduction of new entities in ST&I-based healthcare “or the meaningful collaboration among stakeholders in planning, implementation, and evaluation” needs efficient tools of governance to enable the multi-directional flow of knowledge through  the academic, business and clinical environments [39].

As mentioned above, issues involving the cultural setting of the regimes of knowledge production, technology development, and decision-making in PM and public health can partially help to explain the lack of dialogue between the two approaches. Then, we point to the existence of at least four interpretative societal dimensions that can help move toward a better understanding of this issue: (1) Epistemic attitudes: Public health providers look at improve healthcare access, coverage, and equity at the population level, exploring what makes groups as homogeneous as possible for efficient policy interventions, while PM knowledge-making aims and its stakeholders’ rationales expend resources addressing what is specific of individuals, exploring the complexity of potential healthcare interventions at a molecular-gene level [40]; (2) Communicational: Expert knowledge from PM brings unknown methodologies, tools, and vocabulary from molecular and biomedical sciences to clinicians, increasing uncertainty and creating additional complications in the physician–patient interaction [31], and low ability to avoid inefficient risk communication by governments as we recently experienced in the pandemic [41]; (3) Health policy pragmatism: Health systems have limited resources and time and are surrounded by governmental political interference and change, while PM initiatives are usually too expensive, slow, and constantly underestimate the role of politics in choosing priorities that have nothing to do with what is relevant for science [24]; and (4) Innovation/regulation paradox: Since public health providers have built rigid (and necessary) health surveillance systems and regulatory policies, as well as sophisticated health technology assessment models, PM stakeholders have claimed that it has limited flexibility to let governments adopt new technologies, raising ethical issues, holding back political decisions, and hampering innovation in the public health sector [42].

Since the 2000s, theoretical frameworks have been dedicated to this issue in the literature from the IS. This field still receives limited attention from researchers in public health, but the novel coronavirus crisis may change this reality. Internationally, this field has been integrated with important studies to measure the implications of PM in health research and systems [43]. Eccles and Mittman (2006) define IS as “the scientific study of methods to promote the systematic uptake of research findings and other evidence-based practices into routine practice, and, hence, to improve the quality and effectiveness of health services” [44]. Bauer and colleagues (2015) affirm that “[a]s healthcare systems work under increasingly dynamic and resource-constrained conditions, evidence-based strategies are essential in order to ensure that research investments maximize healthcare value and improve public health. Implementation science (IS) plays a critical role in supporting these efforts” [45].

The dissemination of IS could be one possible outcome of the pandemic for national systems of science, technology, and health. It is important to recognize the need to move forward in new ways to integrate knowledge generated by scientific knowledge production regimes and public health practice [46].

New research has been rapidly emerging as potential solutions to overcome this gap. So-called precision public health (PPH) has been advocated by analysts from PM and health systems around the world [47] as a potential agenda to overcome the lack of dialogue and to balance the asymmetries between the two approaches. However, we will not dedicate time to this topic in this paper, since the aim here is to point out problems raised by the pandemic and understand them from a theoretical interdisciplinary perspective.

## Conclusions

The COVID-19 pandemic presented an important contradiction in health research policies and systems globally: despite the rapid development of technological solutions, PM approaches and those infrastructures did not provide an adequate response to the public health crisis. A gap became apparent between the science and innovation agendas and public health demands, as the production of technologies and delivery of science-based solutions ran in parallel with public sector demands.

A central objective of our paper is to stimulate thinking on how the public relevance of technological development in public health can be understood as a problem of governance and capacity [48]. Institutional change is fundamental to advancing the use of PM for public health in different contexts, with improvement in the collaboration between experts, S&TP, the healthcare industry, and the healthcare system. Kukk and colleagues (2015) advocate institutional change as a crucial component in fostering a favourable environment for technological development, learning, and more resilient collaboration networks between key stakeholders [49]. An analysis of ST&I and regulatory movements in public health is crucial to improving processes of institutional design, that is, strategies and tools of governance of knowledge platforms in healthcare, and its specific historical and cultural contexts [50].

Bottlenecks such as those presented by the pandemic are research findings per excellence of the importance of strengthening the interchange between different theoretical approaches in STS, IS, and PPH. They can help to significantly advance an interdisciplinary academic space for the discussion of technological development approaches and initiatives in public health.

In the sphere of policy-making, the COVID-19 pandemic has led us to reflect on the importance of pursuing political pacts for ST&I in public health that ensures equitable and responsible access to new health technologies to respond to future health crises. The novel coronavirus outbreak has proven that only a collective, interdisciplinary, cross-sectoral, and integrative framework of policies can respond effectively to potential new healthcare emergencies.

Among the main lessons learned from the pandemic experience, we can highlight three claims toward improving the public relevance of ST&I approaches in healthcare in the post-pandemic context. First, the relevant role of policy-makers in integrating S&TP with the public healthcare system’s planning and policies. Before the outbreak, policy-makers were instrumental in translating the potential of S&TP toward efficient governance schemes for science and public health innovation, at the national and international levels. However, over the course of the pandemic, they faced a lack of governance capacity for leading global health programmes and providing accelerated emergency health assistance, as well as for advancing the production of knowledge addressing unmet public health needs.

Secondly, the pandemic led to an optimistic scenario about ST&I and its potential to react to future crises in public health. The learning generated by this experience should serve as a lever for the construction of a more specific system of public policies that takes into account the potential of national actors, and that is capable of effectively seeking complementary expertise to improve mechanisms of management of S&T for public health, both inside and outside the country. This system must always seek to implement measures that guarantee equity in healthcare, even when using PM approaches to offer products and services of higher quality. The promotion of a new deal between industry and society could accelerate this progress toward more well-balanced infrastructural and financial support, both in “research for innovation” and “research for public health”.

Lastly, we learn that continuing the practice of primarily responding to problems is not the most effective way to solve challenges that require planning, management, and preparing for future scenarios. Future preparedness assessment reports and panels must be institutionalized by health systems, and articulated alongside other actors from academia and the health industry, so that responses to future health crises will result in a lower cost of lives and resources.

The COVID-19 pandemic has underscored the existence of a socially responsible and active scientific community and a capillary universal health system. A long-term S&TP must now be better articulated within academia as part of the solution for more equitable knowledge platforms between ST&I landscapes and the health system. The critical assessment and development of (not only) technology-oriented responses are among the first objectives toward achieving results in this direction.

## Data Availability

Not applicable.

## Abbreviations

CDC: Centers for Disease Control and Prevention
COVID-19: Coronavirus disease 2019
ST&I: Science, technology, and innovation
FDA: Food and Drug Administration
ISI WoS: Institute for Scientific Information Web of Science
IS: Implementation sciences
PM: Precision medicine
PMC: Precision medicine coalition
PPH: Precision public health
EXPH: Expert Panel on Effective Ways of Investing in Health
R&D: Research and development
RT-PCR: Reverse transcription polymerase chain reaction
S&TP: Science and technology policy
PMI: Obama Administration’s Precision Medicine Initiative 2015
SARS-CoV-2: Severe acute respiratory syndrome coronavirus 2
EC: European Commission
SUS: Sistema Único de Saúde
STS: Science and technology studies
